# Fasting Plasma Glucose Mediates the Prospective Effect of Maternal Metal Level on Birth Outcomes: A Retrospective and Longitudinal Population-Based Cohort Study

**DOI:** 10.3389/fendo.2021.763693

**Published:** 2021-11-16

**Authors:** Zixing Zhou, Dandan Yu, Gengdong Chen, Pengsheng Li, Lijuan Wang, Jie Yang, Jiaming Rao, Dongxin Lin, Dazhi Fan, Haiyan Wang, Xiaoyan Gou, Xiaoling Guo, Dongmei Suo, Fang Huang, Zhengping Liu

**Affiliations:** ^1^ Foshan Fetal Medicine Research Institute, Foshan Women and Children Hospital Affiliated to Southern Medical University, Foshan, China; ^2^ Department of Obstetrics, Foshan Women and Children Hospital Affiliated to Southern Medical University, Foshan, China

**Keywords:** metal, fasting plasma glucose, birth outcome, mediating effect, cohort study

## Abstract

**Objective:**

Previously, we found that the presence of maternal serum metals before the 24th week of gestation prospectively increased fasting plasma glucose (FPG) at 24–28 weeks. We further explored the prospective association between levels of metals and neonatal outcomes and assessed the mediating effects of FPG on these relationships.

**Methods:**

A total of 7,644 pregnant women were included in a retrospective cohort study, and the relationships between metals [manganese (Mn), copper (Cu), lead (Pb), zinc (Zn), and magnesium (Mg)] and birth outcomes were explored. Quantile and linear regressions were performed to detect the shifts and associations between metals and neonatal size distribution focused on the 10th, 50th, and 90th percentiles. Mediation analysis was performed to assess the mediating effect of FPG on metals and birth outcomes.

**Results:**

After adjustment, a 50% increase in Mn and Zn levels was related to a 0.136-cm (95% CI: 0.067–0.205) and 0.120-cm (95% CI: 0.046–0.193) increase in head circumference, respectively. Based on head circumference distribution, the magnitude of the association with Mn was smaller at the upper tail, while the magnitude of correlation with Zn was greater at the upper tail. A 50% increase in Mn and Zn levels was related to a 0.135-cm (95% CI: 0.058–0.212) and 0.095-cm (95% CI: 0.013–0.178) increase in chest circumference, respectively. The magnitude of the association with Mn increased with increasing chest circumference, while the magnitude of correlation with Zn decreased with increasing chest circumference. FPG explained 10.00% and 17.65% of the associations of Mn with head and chest circumference. A positive indirect effect of Zn associated with head circumference (0.004, 95% CI: 0.002–0.006) and chest circumference (0.005, 95% CI: 0.003–0.008) through FPG was also observed, and the estimated proportion of the mediating effect was 13.79% and 26.32%, respectively.

**Conclusion:**

Maternal serum Mn and Zn levels before the 24th week of gestation may prospectively increase the circumference of the neonatal head and chest. FPG at 24–28 weeks had positive mediating effects on these relationships. Further research is needed to identify a balance between maternal blood glucose and birth size.

## Introduction

Environmental exposure including metals in pregnant women has been associated with health effects in both the mother and her offspring (1, 2). Such metals can enter the maternal body through air, water, food, and other forms of exposure and, thus, have an impact on health (3). Moreover, the metals can have greater health implications on the offspring through the mother as fetal organs and systems are in a critical period of plasticity and sensitivity to the environment (4). Metal exposure has been related to fetal growth in the short term, and it could also result in various health problems in the long term, such as neurodevelopment and cardiovascular disease (5, 6).

Pregnant women are in a critically important state for susceptibility to metal effects because of hemodynamics, hormone changes, and immature immune systems (7), and metals could further affect fetal growth after passing through the placental barrier (8). Studies have found that prenatal exposure to certain ranges of manganese (Mn) (9), copper (Cu) (10), lead (Pb) (11), zinc (Zn) (12), and magnesium (Mg) (13) is linked to adverse birth outcomes, including small-for-gestational-age (SGA) births, low birth weight, and preterm delivery. Similarly, low-level prenatal Pb exposure, as well as elevated Mn and Zn levels, might increase the risk of preterm birth and SGA (14). Moreover, increasing concentrations of Mn have been linked to decreasing birth weight (15). However, the associations between these metals and birth outcomes have been inconsistent. Increasing placental Mn could slightly increase neonatal head circumference in females (16). Pb has been shown to elevate the risk of SGA in infants (11) and was inversely associated with birth weight, birth length, and head circumference in observational studies (17). Furthermore, Mg supplementation during pregnancy might increase birth weight (13). Moreover, prenatal exposure to Pb and Mn has been assessed as showing no relation to birth weight and length (8). Additionally, there seems to be an optimal range of exposure to some metals; for instance, both low and high levels of Mn are associated with lower birth weight and smaller head circumference (18). Meanwhile, an optimum level of Zn and Cu in pregnant women could reduce the risk of low birth weight (19).

Fasting plasma glucose (FPG) is predominantly produced following the decomposition of liver glycogen, while basal insulin secretion can inhibit the output of liver glycogen and prevent higher FPG. Therefore, the level of fasting blood glucose can objectively reflect the secretion level of basal insulin in patients. Elevated metal levels can accumulate in pancreatic tissue where they may contribute to insulin resistance or damage islet β cells and impair insulin secretion (20). Mn has been shown to inhibit glucose-stimulated insulin secretion in β cells (21), while higher urinary Zn or Pb could increase FPG of Chinese adults (22). In addition, serum Mg levels have been negatively associated with fasting insulin, and higher copper concentrations could increase the risk of glucose dysregulation during pregnancy (23, 24). Our previous study (25) also found that metals including Mn, Cu, Pb, Zn, and Mg in early pregnancy were prospectively related to later FPG in pregnant women. Moreover, high FPG levels have been linked to macrosomia and large-for-gestational-age infants (26). It has also been revealed that correlations between postprandial blood glucose and primary outcomes are weaker than for FPG (27). Nevertheless, we found no relevant studies that clarify the relationship among metals, FPG, and birth outcomes, especially in a longitudinal direction of complete pregnancy.

Therefore, after combining our previous study with those of others, we hypothesized that earlier metal exposure in pregnant women was prospectively associated with final birth outcome, and later FPG mediated their relationship. Here, we tested this hypothesis in a retrospective and longitudinal population-based cohort study to explore i) whether maternal early metal exposure including Mn, Cu, Pb, Zn, and Mg before 24 weeks was related to birth outcomes and ii) whether a maternal FPG of 75 g oral glucose tolerance test (OGTT) at 24–28 weeks mediated the relationship between metal exposure and birth outcomes.

## Methods

### Study Population

The retrospective cohort study was conducted between January 2017 and December 2018 in Foshan, China. Pregnant women were recruited at the first obstetric clinic visit in the Southern Medical University Affiliated Foshan Women and Children Hospital. The study was approved by the Human Subjects Committee of our hospital. The inclusion criteria were as follows: 1) singleton pregnancy, 2) no diabetes prior to pregnancy, and 3) gestational age in the first trimester (<14 weeks). The exclusion criteria were as follows: 1) no available data on metal detection before 24 weeks of pregnancy (<24 weeks), 2) missing data of OGTT at 24–28 weeks of gestation, and 3) stillbirth or without delivery data in our hospital. Of 11,845 women who initially met the above inclusion criteria, 7,646 were ultimately included for the study (**Figure S1**).

### Data Collection and Definition of Variables

Medical data were collected from hospital computerized databases or clinical charts. The information of maternal demographic characteristics included maternal age, employment status, maternity insurance, family history of diabetes, family history of hypertension, smoking habit, and drinking habit. The clinical history information included parity, and prenatal visit records consisted of gestational age and BMI at the first prenatal examination in the first trimester; gestational age for metal detection; serum levels of the metals Mn, Cu, Pb, Zn, and Mg; OGTT results at 24–28 weeks; diagnosis of gestational diabetes mellitus (GDM) and hypertensive disorders of pregnancy (HDP); and weight gain during the whole pregnancy. The delivery records (e.g., gestational age at delivery, neonatal sex, birth weight, birth length, head and chest circumference) were retrieved. All identifying patient information was anonymized to protect patient identity.

The diagnostic criteria for GDM referred to the criteria of the International Association for Diabetes in Pregnancy Study Group (IADPSG): pregnant women were given OGTT at 24–28 weeks. The results were classified based on one of the following cutoff points: fasting plasma glucose (FPG, OGTT0) ≥5.1 mmol/L or 1-h PG (1-h postprandial plasma glucose, OGTT1) ≥10.0 mmol/L or 2-h PG (2-h postprandial plasma glucose, OGTT2) ≥8.5 mmol/L (28). First-trimester BMI (kg/m^2^) was divided into categories according to Chinese BMI criteria for general adults: BMI < 18.5 kg/m^2^, 18.5 kg/m^2^ ≤ BMI < 24 kg/m^2^, 24 kg/m^2^ ≤ BMI < 28 kg/m^2^, and BMI ≥ 28 kg/m^2^ (29). Hypertensive disorders of pregnancy (HDP) included gestational hypertension, preeclampsia, eclampsia, superimposed preeclampsia on chronic hypertension, and chronic hypertension in pregnancy (30).

### Measurement of Serum Metal Concentration

Metal detection was performed according to the methods described in our previous article (25). Maternal serum metal concentration was assessed only once before 24 weeks of pregnancy in our hospital. Peripheral venous blood samples (2 ml) were drawn in the obstetric clinic and then transported to the Department of Laboratory Medicine of the hospital within 1 h for serum metal detection, using the polarography method (AS-9000 C, AWSA, Wuhan, China). The detection limit of the polarographic channel was ≤1 × 10^−8^ mol/L. All of the metal measurements were above the limit. The coefficient of variation (CV) of detection was ≤1%, and the relative error (B) of detection was ≤1%.

### Statistical Analysis

Baseline characteristics were described as mean ± SD or number (%). In the analysis of birth outcomes, maternal serum metal levels were categorized into three parts according to tertiles. Comparisons for birth outcomes among three groups of metals used ANOVA, and the pairwise comparisons were conducted using Dunnett with a control category of the 1st tertile. The analyses were conducted using SPSS 24.0 software.

Quantile regression was performed to detect the distribution shifts of neonatal size (e.g., head circumference) and to explore the associations with metal levels primarily occurring at the tails of neonatal size. In particular, the 10th, 50th, and 90th percentiles of neonatal size were focused on. The metal linear regression was established for each percentile and the mean of neonatal size. Metal level was log-transformed based on a 1.5 logarithm function, assuring that 1-unit change on the transformed scale was close to or within the IQR. One-unit difference on the natural log-transformed scale corresponded to a much wider range than the IQR on the original range (24). The models were adjusted for the following factors: maternal age, employment status, parity, maternity insurance, family history of diabetes, family history of hypertension, HDP, GDM, BMI, gestational age for metal detection, weight gain during pregnancy, infant sex, and gestational age at delivery. These analyses were performed in R 3.5.2, using the packages quantreg and forestplot in quantile regression.

In our previous study (25), we found significant associations between Mn, Cu, Pb, Zn, and Mg and OGTT0. Thus, we aimed to further explore the mediating effect of OGTT0 on metals and birth outcomes. Mediation analysis, one model of the structural equation modeling, was performed using the robust maximum-likelihood estimation. Standardized coefficients of direct, indirect, and total effects were estimated, and the 95% CI was measured using the bootstrap method with 2,000 resamplings. The model was adjusted for the following factors: maternal age, employment status, parity, maternity insurance, family history of diabetes, family history of hypertension, HDP, BMI, gestational age for metal detection, weight gain during pregnancy, infant sex, and gestational age at delivery. The mediation proportion of indirect effect was calculated as follows: Estimated mediated = Indirect effect/Total effect × 100%. Total effect = Direct effect + Indirect effect. The data were analyzed using Mplus 7.4 (Muthén and Muthén). A two-sided *P*-value <0.05 was considered to indicate statistical significance.

## Results

### Baseline Characteristics

**Tables 1** and **S1** show the characteristics of our study population. The mean maternal age was 30.13 years. Of the pregnant participants, 55.45% were employed and 67.51% of those had maternity insurance. None of the participants had smoking or drinking habits. Only 0.47% of women had a family history of diabetes. However, the incidence of GDM was 15.93%. The mean BMI in the first trimester was 21.38 kg/m^2^, and the mean weight gain during the whole of pregnancy was 12.70 kg. The mean gestational age at delivery was 38.47 weeks. The mean birth weight, birth length, head circumference, and chest circumference of the newborns were 3,155.13 g, 49.12 cm, 32.89 cm, and 32.70 cm, respectively.

**Table 1:** The baseline characteristics of the study population in the cohort study.

Characteristics	Total (*n* = 7,646)
Maternal characteristics	
Maternal age (mean ± SD, years)	30.13 ± 4.70
Age < 30 (*n*, %)	3,749 (49.03)
30 ≤ age < 35 (*n*, %)	2,556 (33.43)
Age ≥ 35 (*n*, %)	1,341 (17.54)
Employment status (*n*, %)	
Unemployed	3,406 (44.55)
Employed	4,240 (55.45)
Parity (*n*, %)	
0	3,685 (48.20)
1	3,726 (48.73)
≥2	235 (3.07)
Maternity insurance (*n*, %)	5,162 (67.51)
Family history of diabetes (*n*, %)	36 (0.47)
Family history of hypertension (*n*, %)	31 (0.41)
Smoking habit (*n*, %)	0
Drinking habit (*n*, %)	0
GDM (*n*, %)	1,218 (15.93)
HDP (*n*, %)	130 (1.70)
First prenatal examination in the first trimester	
Gestational age (mean ± SD, weeks)	11.13 ± 1.55
BMI (mean ± SD, kg/m^2^)	21.38 ± 3.09
BMI < 18.5 (*n*, %)	1,212 (15.85)
18.5 ≤ BMI < 24 (*n*, %)	5,107 (66.79)
24 ≤ BMI < 28 (*n*, %)	1,067 (13.96)
BMI ≥ 28 (*n*, %)	260 (3.40)
Gestational age for metal detection (mean ± SD, weeks)	16.56 ± 2.94
OGTT (mean ± SD, mmol/L)	
OGTT0	4.27 ± 0.38
OGTT1	7.82 ± 1.65
OGTT2	6.80 ± 1.40
Weight gain during pregnancy (kg)	12.70 ± 4.02
Birth characteristics	
Female (*n*, %)	3,595 (47.02)
Gestational age at delivery (mean ± SD, weeks)	38.47 ± 1.45
Birth weight (mean ± SD, g)	3,155.13 ± 436.44
Birth length (mean ± SD, cm)	49.12 ± 2.03
Head circumference (mean ± SD, cm)	32.89 ± 1.50
Chest circumference (mean ± SD, cm)	32.70 ± 1.81

HDP, hypertensive disorders of pregnancy; GDM, gestational diabetes mellitus; SD, standard deviation; BMI, body mass index; OGTT, oral glucose tolerance test; OGTT0, fasting plasma glucose; OGTT1, 1-h postprandial plasma glucose; OGTT2, 2-h postprandial plasma glucose.

### Distribution of Metals and the Association With Birth Outcomes

The mean gestational age of metal detection was 16.56 weeks (**Table 1**). **Table S2** shows the geometric mean and percentiles of the levels of five metals. Results from metals classified into tertiles indicated that head circumference in the second and third tertiles of Mn or Zn was significantly larger than that in the first tertile (*P* < 0.05) (**Table 2**). Chest circumference in the second and third tertiles of Mn was also significantly larger than that in the first tertile (*P* < 0.05). No significant difference of gestational age, birth weight, or birth length was observed among the three tertiles of all five metals.

**Table 2:** Association between metal concentrations and birth outcomes based on three different metal levels.

Metals	GA (weeks)	*P*-value^a^	BW (g)	*P*-value^a^	BL (cm)	*P*-value^a^	HC (cm)	*P*-value^a^	CC (cm)	*P*-value^a^
Mn (μmol/L)		0.977		0.801		0.504		<0.001**		0.014*
1st tertile (≤0.78)	38.56 ± 1.41	Ref	3,150.07 ± 432.22	Ref	49.08 ± 1.97	Ref	32.79 ± 1.45	Ref	32.62 ± 1.75	Ref
2nd tertile (0.79–0.86)	38.57 ± 1.50	0.996	3,158.07 ± 440.76	0.762	49.13 ± 2.09	0.574	32.94 ± 1.52	0.001*	32.73 ± 1.84	0.052
3rd tertile (≥0.87)	38.57 ± 1.44	0.968	3,155.89 ± 436.44	0.823	49.14 ± 2.03	0.440	32.93 ± 1.52	0.002*	32.76 ± 1.894	0.011*
Cu (μmol/L)		0.081		0.564		0.383		0.665		0.105
1st tertile (≤18.00)	38.62 ± 1.39	Ref	3,158.68 ± 435.14	Ref	49.16 ± 1.98	Ref	32.90 ± 1.48	Ref	32.75 ± 1.79	Ref
2nd tertile (18.01–23.70)	38.55 ± 1.51	0.170	3,147.66 ± 438.10	0.511	49.10 ± 2.08	0.409	32.90 ± 1.50	0.998	32.70 ± 1.83	0.522
3rd tertile (≥23.71)	38.53 ± 1.44	0.062	3,158.06 ± 436.16	0.987	49.09 ± 2.04	0.357	32.86 ± 1.52	0.632	32.64 ± 1.81	0.063
Pb (μg/L)		0.953		0.481		0.421		0.572		0.381
1st tertile (≤19.60)	38.57 ± 1.44	Ref	3,151.82 ± 432.40	Ref	49.14 ± 2.00	Ref	32.91 ± 1.50	Ref	32.74 ± 1.80	Ref
2nd tertile (19.61–34.00)	38.56 ± 1.46	0.939	3,149.96 ± 434.40	0.983	49.14 ± 2.01	1.000	32.89 ± 1.49	0.919	32.68 ± 1.81	0.410
3rd tertile (≥34.01)	38.57 ± 1.45	0.997	3,163.60 ± 442.05	0.526	49.07 ± 2.09	0.421	32.89 ± 1.51	0.478	32.68 ± 1.82	0.350
Zn (μmol/L)		0.923		0.990		0.874		0.006*		0.117
1st tertile (≤107.80)	38.56 ± 1.41	Ref	3,152.55 ± 430.64	Ref	49.10 ± 1.97	Ref	32.81 ± 1.44	Ref	32.64 ± 1.75	Ref
2nd tertile (107.81–120.00)	38.58 ± 1.49	0.891	3,154.71 ± 437.72	1.000	49.13 ± 2.03	0.838	32.93 ± 1.51	0.007*	32.72 ± 1.83	0.177
3rd tertile (≥120.01)	38.57 ± 1.45	0.973	3,256.12 ± 441.13	0.988	49.12 ± 2.03	0.900	32.92 ± 1.54	0.016*	32.70 ± 1.81	0.101
Mg (mmol/L)		0.809		0.976		0.304		0.045*		0.026*
1st tertile (≤1.31)	38.56 ± 1.41	Ref	3,154.41 ± 425.44	Ref	49.11 ± 1.98	Ref	32.90 ± 1.47	Ref	32.72 ± 1.77	Ref
2nd tertile (1.32–1.50)	38.58 ± 1.44	0.860	3,154.32 ± 443.57	1.000	49.17 ± 2.00	0.455	32.93 ± 1.48	0.694	32.75 ± 1.80	0.762
3rd tertile (≥1.51)	38.56 ± 1.49	0.977	3,156.71 ± 440.54	0.974	49.08 ± 2.11	0.873	32.83 ± 1.55	0.150	32.62 ± 1.87	0.086

GA, gestational age; BW, birth weight; BL, birth length; HC, head circumference; CC, chest circumference; Mn, manganese; Cu, copper; Pb, lead; Zn, zinc; Mg, magnesium.

*P < 0.05, **P < 0.001. Ref, reference; Bold value, P < 0.05.

a One-way ANOVA and pairwise comparisons using Dunnett with a control category of the 1st tertile.

**Figure 1:**
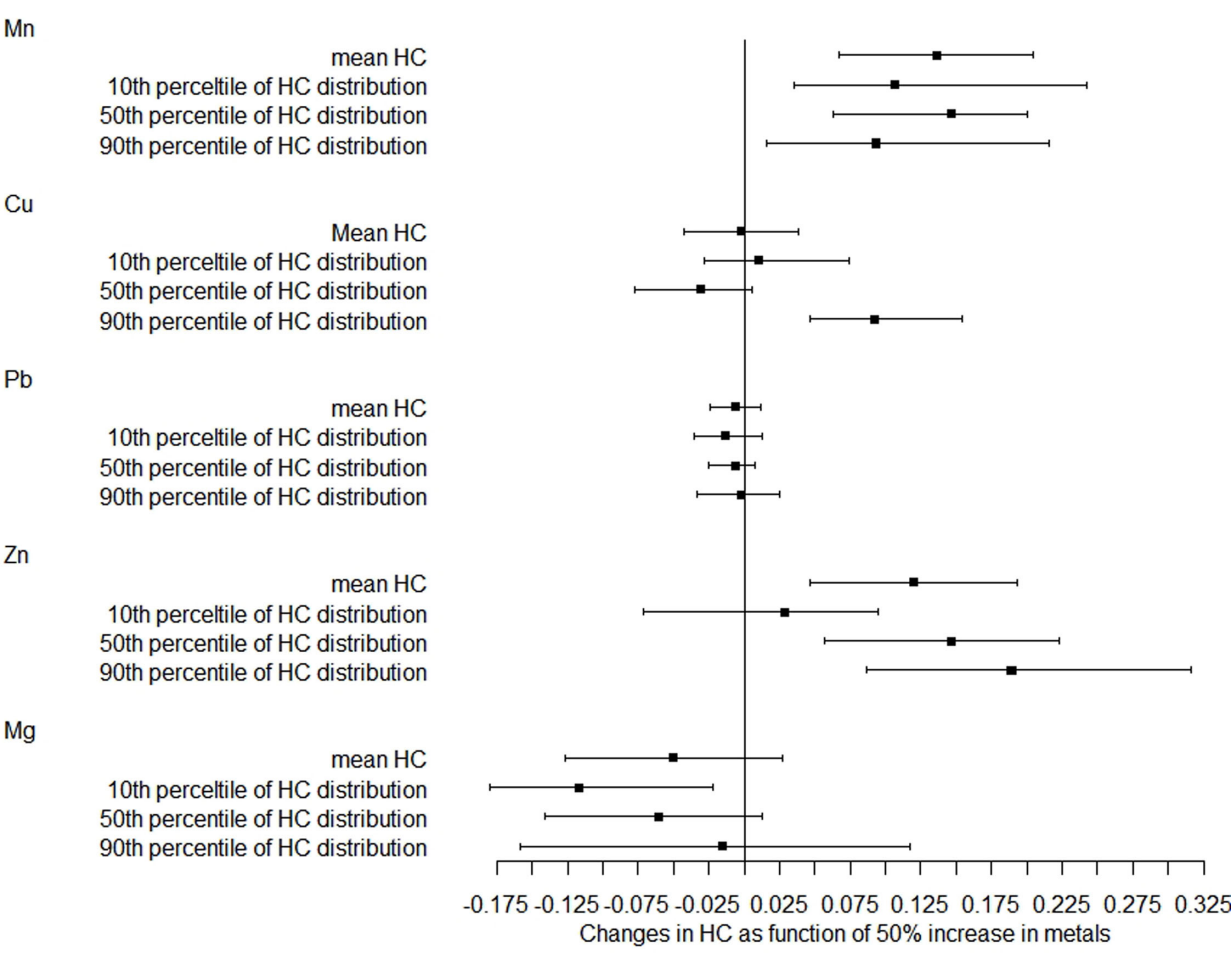
Distribution changes in head circumference as a function of 50% increase in metals. The models were adjusted for maternal age, employment status, parity, maternity insurance, family history of diabetes, family history of hypertension, HDP, GDM, BMI, gestational age for metal detection, weight gain during pregnancy, infant sex, and gestational age at delivery. Mn, manganese; Cu, copper; Pb, lead; Zn, zinc; Mg, magnesium; HC, head circumference.

In the further analysis, we explored the associations between metals and the distribution shifts of head and chest circumference. As shown in **Figure 1**, a 50% increase in Mn and Zn levels was related to a 0.136-cm (95% CI: 0.067–0.205) and 0.120-cm (95% CI: 0.046–0.193) increase in mean head circumference after adjustment, respectively. Based on the 10th, 50th, and 90th percentiles of head circumference distribution, the magnitude of the association with Mn was smaller at the upper tail, while the magnitude of correlation with Zn was greater at the upper tail. However, no significant association was found between Pb, Cu, and Mg levels and head circumference distribution.

**Figure 2:**
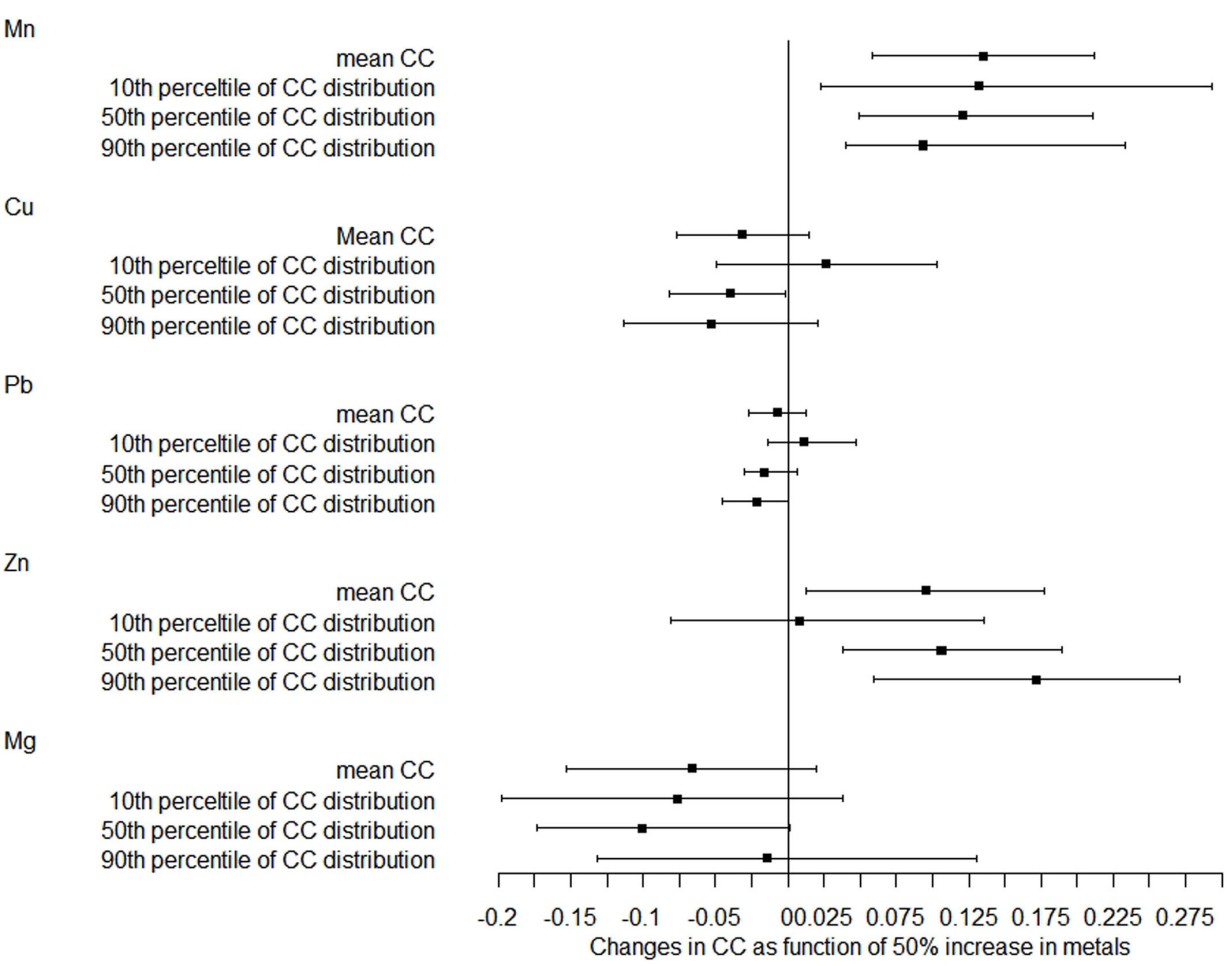
Distribution changes in chest circumference as a function of 50% increase in metals. The models were adjusted for maternal age, employment status, parity, maternity insurance, family history of diabetes, family history of hypertension, HDP, GDM, BMI, gestational age for metal detection, weight gain during pregnancy, infant sex, and gestational age at delivery. Mn, manganese; Cu, copper; Pb, lead; Zn, zinc; Mg, magnesium; CC, chest circumference.

The correlations of metals with chest circumference were similar to those of head circumference (**Figure 2**). After adjustment, a 50% increase in Mn and Zn levels was related to a 0.135-cm (95% CI: 0.058–0.212) and 0.095-cm (95% CI: 0.013–0.178) increase in mean chest circumference, respectively. The magnitude of the association with Mn was increased with the increase of the 10th, 50th, and 90th percentiles of chest circumference distribution, while the magnitude of correlation with Zn was decreased with the increase of the distribution shifts. There was also no relationship between Pb, Cu, and Mg concentrations and chest circumference distribution.

### Mediating Effects of OGTT0 on Metals and Head and Chest Circumference

**Figure 3:**
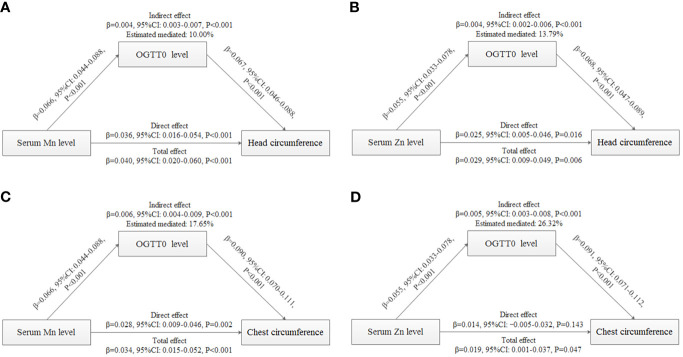
Mediation analysis with standardized coefficients of metals, OGTT0, and birth outcomes. **(A)** Analysis of serum Mn level, OGTT0 level, and head circumference. **(B)** Analysis of serum Zn level, OGTT0 level, and head circumference. **(C)** Analysis of serum Mn level, OGTT0 level, and chest circumference. **(D)** Analysis of serum Zn level, OGTT0 level, and chest circumference. The model was adjusted for maternal age, employment status, parity, maternity insurance, family history of diabetes, family history of hypertension, HDP, BMI, gestational age for metal detection, weight gain during pregnancy, infant sex, and gestational age at delivery. All models were found to be statistically significant (*P* < 0.001).

Because of the significant correlations of Mn/Zn and head/chest circumference, mediation analysis was performed to explore the mediating effects of OGTT0 (**Figure 3**). After controlling for the influence of covariates, the results showed that OGTT0 level explained 10.00% (indirect effect: 0.004, 95% CI: 0.003–0.007, *P* < 0.001) and 17.65% (indirect effect: 0.006, 95% CI: 0.004–0.009, *P* < 0.001) for the associations of serum Mn concentration with head and chest circumference. A positive indirect effect of Zn association with head circumference (0.004, 95% CI: 0.002–0.006, *P* < 0.001) and chest circumference (0.005, 95% CI: 0.003–0.008, *P* < 0.001) through OGTT0 level was also observed, and the estimated proportions of the mediating effect were 13.79% and 26.32%, respectively. In conclusion, there were positive correlations between maternal serum Mn and Zn levels and neonatal head and chest circumference, and these positive correlations were mediated by OGTT0 glucose level.

## Discussion

The current study showed that maternal serum Mn and Zn levels before 24 weeks of gestation could prospectively increase neonatal head and chest circumference. For the head circumference, the magnitude of the association with Mn was smaller at the upper tail, while the magnitude of correlation with Zn was greater at the upper tail. For the chest circumference, the magnitude of the association with Mn was greater at the upper tail, while the magnitude of correlation with Zn was smaller at the upper tail. In addition, FPG at 24–28 weeks mediated the positive effect of Mn or Zn exposure on head or chest circumference. No relationships between Mn and Zn and other birth outcomes including gestational age, birth weight, and birth length were found. No correlation between Pb, Cu, and Mg and birth outcomes was observed.

To date, several studies have explored the association between Mn exposure and birth outcome. However, only a few articles report a prospective and positive relationship between early maternal Mn level and neonatal birth size and the mediating effect of FPG on them. Maternal blood Mn has previously been shown to be positively related to neonatal head circumference, but not to birth weight and birth length (31), which is consistent with our findings. However, our present results were contrary to some other published studies, as follows: maternal Mn level in erythrocytes in the second trimester has been demonstrated to be negatively linked to birth weight and head and chest circumferences (32). This might have contributed to a slightly higher exposure to environmental Mn among pregnant women living in this region (geometric mean Mn in our study: 0.81 μmol/L vs. 0.72 μmol/L in theirs). Furthermore, elevated umbilical cord blood Mn may decrease birth weight, but was not related to head circumference and birth length (15). Moreover, maternal serum Mn level was not associated with birth weight, but was negatively linked to head circumference (33). Although some studies have also found a positive correlation between metals and birth size, the results were different from ours. A previous report described that maternal Mn was related to higher birth length (34). Also, maternal hair but not blood Mn concentrations showed a positive linear association with infant chest circumference (35). This may be because the amount of Mn might vary in different parts of the body, leading to different correlations between Mn and birth outcomes. In addition, considering that the rapid increase in fetal body weight during late pregnancy is associated with an accelerated growth of fetal peripheral muscle and fat deposition, neonatal birth weight might be more affected by the intrauterine environment, including nutritional supplementation from the mother during pregnancy, than neonatal head and chest circumference (36). Neonatal head and chest growth might be more susceptible to metal exposure in early pregnancy than birth weight and length; however, birth weight and length might be more susceptible to the combined effects of maternal nutrition and maternal and paternal height and genes in late pregnancy. We cautiously assumed that the combined effects in late pregnancy might be stronger than the effect of Mn in early pregnancy and, thus, masked the effect of metals. In future studies, it will be important to further explore the relationship between neonatal birth size and metals by integrating nutrition, genes, and other factors during pregnancy.

The results of the current study further showed that maternal Mn might affect the birth outcome by influencing FPG during pregnancy. Mn, both an essential element and a potential toxicant, is a component of several enzymes and an activator for enzymes. Deficiency of Mn might affect growth and development of the body and glucose metabolism, and excessive intake of Mn could cause poisoning (37). Mn is suggested to inhibit glucose-stimulated insulin secretion in β cells by impairing mitochondrial function (21). One population-based study has shown a positive relationship of urinary Mn with hyperglycemia risk in Chinese coke oven workers (38). Meanwhile, our previous study found that early maternal serum Mn could increase FPG at 24–28 weeks (25).

Larger offspring size has been attributed mainly to higher pregnancy glucose levels. We made a cautious hypothesis that the long-term effects of fetal glucose exposure could affect fetal β-cell capacity, especially exposure to higher FPG, and thus, higher maternal FPG might indirectly affect neonatal birth outcomes. Therefore, FPG might mediate the effect of Mn level on birth outcome. However, there are currently no metal requirement guidelines for pregnant women. The Mn level of our study population was slightly higher than that found in other populations, and serum Mn levels in most pregnant women in our study were below the lower limit of the reference range (0.15–0.22 μmol/L) for healthy adults (39). Mn requirements for pregnant women greatly increase as the fetus grows (40); however, this high demand might impair the function of the islets and cause elevated blood glucose. Further research is needed to investigate the balance between maternal blood glucose and neonatal size.

Zn is a component of zinc-containing metalloenzymes involved in the activity of more than 80 enzymes. Zn is also an important immunomodulator and growth cofactor and plays an important role in anti-oxidation, anti-apoptosis, and anti-inflammation. About 90% of Zn in the body is distributed in muscles and bone (19). Blood Zn concentration is about 0.1~0.15 mmol/L in the general population (41), and our study population showed similar levels. Previous studies have reported that higher Zn levels in early pregnancy could increase head circumference (34), and prenatal Zn has been associated with greater chest circumference (42), which are consistent with our findings. Zn supplementation is also beneficial to head growth rate in infants (43). However, one report also states that maternal Zn level was inversely related to birth weight (12). It could be argued that the discordant results are owing to the different levels of original Zn exposure and different study populations with different underlying diseases affecting fetal growth. Head circumference in early life is an important indicator of brain development. Furthermore, it could affect neuronal growth and brain function (19). Brain development is incredibly complex and takes place in two major stages: the first 20 weeks involve fetal organogenesis and neurogenesis, and during weeks 20–40, there is continued neuronal growth, neural migration, and maturation. Brain development is very rapid, from 32 to 39 weeks when the brain can come to weigh 30% as much as an adult (44). In our study, metal exposure before 24 weeks of gestation had significant implications for the two stages of fetal brain development. Rapid brain growth increases vulnerability to unfavorable environmental conditions. In our study, although the head circumference increased slightly with the increase of Zn concentration, it also had some clinical significance. Large population studies are needed to explore the relationship between small differences in neonatal head circumference and brain function. In addition, Zn and Mn were related to birth head and chest circumference but had no relationship with birth weight and length. This might be associated with neonatal birth size as Zn has the same effect as described for Mn above. Zn is also an essential trace element for insulin synthesis, storage, and release (45). However, excess levels of Zn during embryogenesis could disturb biochemical processes and could be teratogenic or ultimately fatal (19). We found that Zn prospectively increased late second-trimester FPG in our previous study (25) and Zn could further increase the head and chest circumference through higher levels of FPG. The relationship of Zn among the three (Zn, FPG, and birth outcome) might be similar to that of Mn. These findings require further epidemiological and molecular studies in both the fetal stage and the childhood stage.

Our current study found no association between Pb and birth outcomes, which is consistent with a previous study (15). However, Pb has been reported to decrease birth weight and head circumference following fetal lead exposure during pregnancy and lactation (46, 47). The normal range of Pb in the general population is <50 μg/L (48), and the Pb levels of our participants were within the normal range. Therefore, Pb level in the normal range might not be associated with birth outcomes, but higher levels above the normal range might have toxic effects on neonatal birth size.

Excess Cu is harmful to human health, but it is rarely excessive in the general population (49), and the demand for Cu is significantly increased in pregnant women (19). Cu deficiency during pregnancy might result in oxidative stress that can reduce fetal growth (19). Serum Cu <350 μg/L (5.51 μmol/L) is linked to conditions of Cu deficiency (50). The minimum concentration in our study population was 7.60 μmol/L. Therefore, we speculated with caution that a significant association between maternal Cu exposure and adverse birth outcomes might not be observed at normal concentrations. The underlying mechanisms need further exploration.

The reference range for serum Mg in adults is 0.75 mmol/L (5% CI: 0.45, 1.05) (51); our study population had a slightly higher level. There is an increasing need for Mg throughout pregnancy, and Mg supplementation might have neuroprotective effects on preterm infants (52). However, limited studies have reported the association between Mg and birth size. Most prenatal Mg supplementation is given in the third trimester, in order to protect neurologic function in preterm infants. Although we found that Mg in early pregnancy was not associated with birth outcome, an appropriate Mg range should be established for pregnant women. Further studies are needed to determine whether Mg in early pregnancy is prospectively related to neurodevelopment of newborns in the long term.

The current study was the first study to show that metal exposure in early pregnancy could affect FPG in the late second trimester and, thus, affect neonatal head and chest circumference. This information may promote new regimens for the personalized control and management of birth size during pregnancy. The study sample size was also relatively large, which increased the accuracy of the findings and made them more applicable to other populations. However, some limitations in the study should be noted. First, despite the large sample size, none of the pregnant women in our study had a smoking or drinking habit. This limits the value of the study. Smoking and drinking habits were investigated in each pregnant woman during prenatal visits as they are risk factors for adverse pregnancy and birth outcomes, not just for the birth size of the newborn. This could be a good habit of women in southern China, although it is impossible to rule out a few cases of concealment. Second, our research was a single-center study; multicenter studies are needed in the future to make the results more precise and reliable. Third, data related to trace element supplementation during pregnancy in this study were not available, which might be a confounding factor affecting the results. We are currently conducting a prospective birth cohort study, aiming to collect more detailed and accurate data. Fourth, the study only examined maternal metal exposure in early pregnancy before 24 weeks, not in the third trimester, placenta, or umbilical cord blood. Metal requirements for pregnant women might vary in different trimesters, and the placental barrier might support different metal effects between mother and offspring. Further epidemiological and molecular studies should be conducted.

In conclusion, our study suggests that maternal serum Mn and Zn levels in early pregnancy may prospectively increase neonatal head and chest circumference. FPG in the late second trimester could positively mediate the association of Mn and Zn exposure with head or chest circumference. However, maternal Mn and Zn levels in early pregnancy were not associated with birth weight and birth length. Further studies should explore the relationship between neonatal birth size and metals by integrating maternal nutrition, living habits, the genes of both parents, and other factors throughout pregnancy. In addition, further research is needed to find a balance between maternal blood glucose and neonatal birth size; the corresponding epidemiological and molecular studies need to explore and reveal the molecular mechanisms.

## Data Availability Statement

The raw data supporting the conclusions of this article will be made available by the authors, without undue reservation.

## Ethics Statement

The studies involving human participants were reviewed and approved by the Ethics Committee of the Southern Medical University Affiliated Foshan Women and Children Hospital (FSFYMEC-2019-024). Written informed consent for participation was not required for this study in accordance with the national legislation and the institutional requirements.

## Author Contributions

ZZ, DS, FW and ZL conceived and designed the study. ZZ, DY, GC, PL, LW, JY, DL, JR, DF, XG, HW and XG carried out data collection. ZZ carried out the statistical analyses and drafted the manuscript. All authors contributed to the article and approved the submitted version.

## Funding

This work was supported by the Basic and Applied Basic Research Foundation of Guangdong Province (Nos. 2019A1515110163 and 2019A1515111011), the Medical Science and Technology Foundation of Guangdong Province (No. C2019090), the Medical Research Foundation of Guangdong Province (No. A2019214), and the Foundation of Science and Technology Agency of Foshan City (Nos. 1920001000248, 1920001000294, and 20200027).

## Conflict of Interest

The authors declare that the research was conducted in the absence of any commercial or financial relationships that could be construed as a potential conflict of interest.

## Publisher’s Note

All claims expressed in this article are solely those of the authors and do not necessarily represent those of their affiliated organizations, or those of the publisher, the editors and the reviewers. Any product that may be evaluated in this article, or claim that may be made by its manufacturer, is not guaranteed or endorsed by the publisher.

## Supplementary Material

Supplementary Figure 1 Follow charts of study population.
