# Supplementary figures and images for: Fasting Plasma Glucose Mediates the Prospective Effect of Maternal Metal Level on Birth Outcomes: A Retrospective and Longitudinal Population-Based Cohort Study
Source: Front Endocrinol (Lausanne). 2021 Nov 16;12:763693. doi: 10.3389/fendo.2021.763693 (PMC8635137; doi:10.3389/fendo.2021.763693)

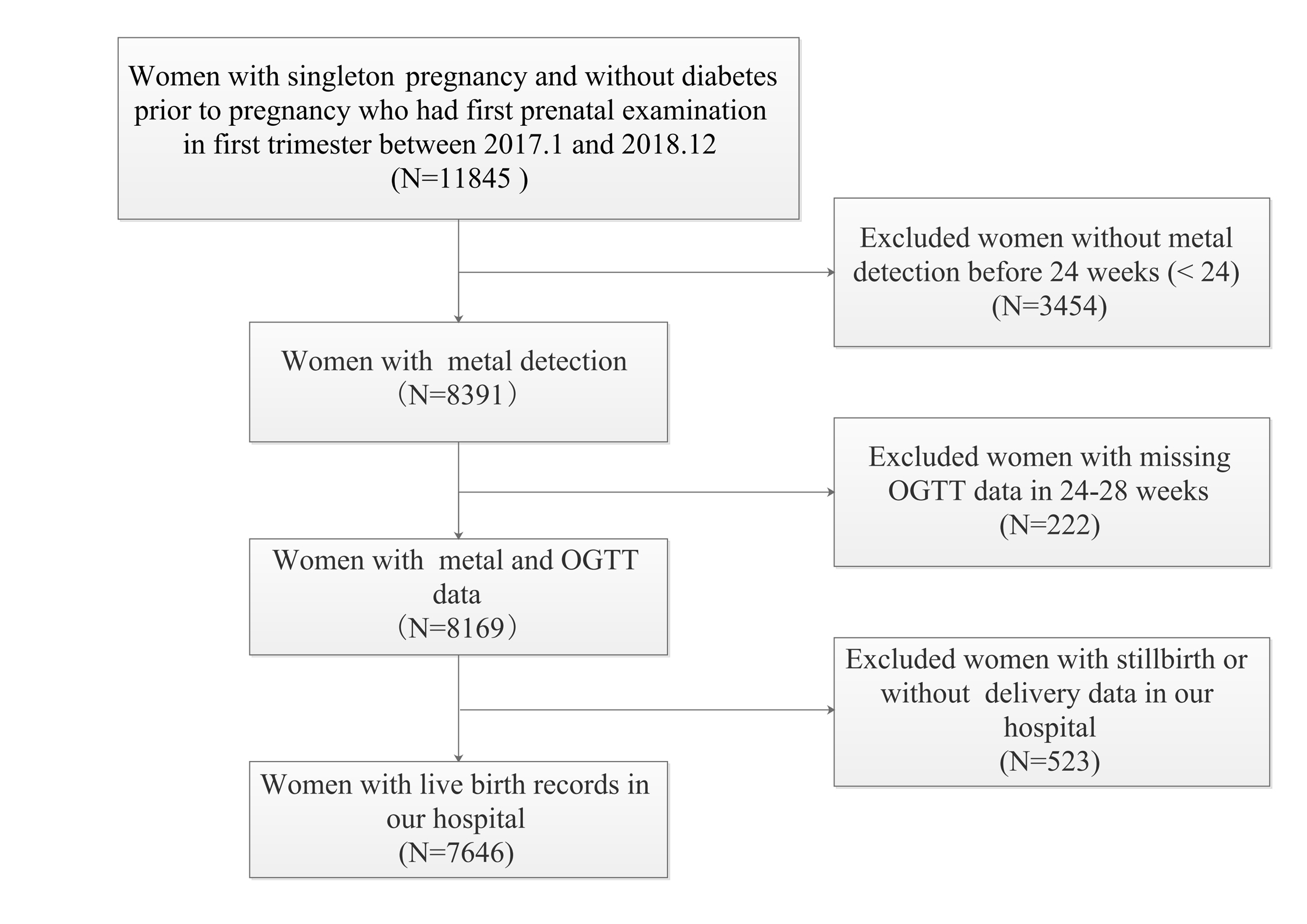

Supplement: Supplementary Figure 1 — Follow charts of study population. [file Image_1.jpeg]
