# Supplementary material for: Fasting Plasma Glucose Mediates the Prospective Effect of Maternal Metal Level on Birth Outcomes: A Retrospective and Longitudinal Population-Based Cohort Study
Source: Front Endocrinol (Lausanne). 2021 Nov 16;12:763693. doi: 10.3389/fendo.2021.763693 (PMC8635137; doi:10.3389/fendo.2021.763693)
Supplement: Supplementary file 2 [file Table_1.docx]

**Table S1** Percentage of gestational diabetes mellitus in different age groups

| **Age groups** | **Total (n = 1218)** |
| --- | --- |
| Age < 30 years old (n, %) | 395 (32.43) |
| 30 ≤ Age < 35 years old (n, %) | 453 (37.19) |
| Age≥ 35 years old (n, %) | 370 (30.38) |

**Table S2** Distributions of serum metal level of study population in different maternal age groups

| Metal | Geometric mean | Min | Max | Percentiles | | | | |
| --- | --- | --- | --- | --- | --- | --- | --- | --- |
|  |  |  |  | 5th | 25th | 50th | 75th | 95th |
| Mn (μmol/L) | 0.81 | 0.03 | 1.15 | 0.60 | 0.75 | 0.82 | 0.89 | 1.01 |
| Age < 30 years old | 0.81 | 0.04 | 1.15 | 0.60 | 0.75 | 0.82 | 0.89 | 1.01 |
| 30 ≤ Age < 35 years old | 0.80 | 0.03 | 1.15 | 0.59 | 0.75 | 0.82 | 0.89 | 1.02 |
| Age ≥ 35 years old | 0.81 | 0.34 | 1.14 | 0.61 | 0.75 | 0.82 | 0.90 | 1.00 |
| Cu (μmol/L) | 20.07 | 7.60 | 32.80 | 11.60 | 16.60 | 20.60 | 25.20 | 30.40 |
| Age < 30 years old | 20.07 | 8.10 | 32.70 | 11.60 | 16.50 | 20.60 | 25.40 | 30.40 |
| 30 ≤ Age < 35 years old | 20.02 | 8.20 | 32.70 | 11.70 | 16.70 | 20.50 | 25.00 | 30.40 |
| Age ≥ 35 years old | 20.14 | 7.60 | 32.80 | 11.40 | 16.70 | 20.70 | 25.50 | 30.59 |
| Pb (μg/L) | 25.23 | 1.10 | 265.00 | 8.60 | 16.70 | 25.20 | 40.00 | 70.17 |
| Age < 30 years old | 24.84 | 1.30 | 265.00 | 8.45 | 16.50 | 25.00 | 38.95 | 70.00 |
| 30 ≤ Age < 35 years old | 25.69 | 1.10 | 198.40 | 8.60 | 17.20 | 25.30 | 41.20 | 72.08 |
| Age ≥ 35 years old | 25.45 | 1.10 | 132.50 | 8.71 | 16.90 | 25.90 | 41.70 | 68.80 |
| Zn (μmol/L) | 113.49 | 50.40 | 219.50 | 86.30 | 104.40 | 113.80 | 124.20 | 145.63 |
| Age < 30 years old | 113.46 | 52.50 | 219.50 | 87.10 | 104.40 | 113.80 | 124.10 | 144.70 |
| 30 ≤ Age < 35 years old | 113.46 | 58.80 | 217.80 | 84.87 | 104.23 | 113.50 | 124.00 | 147.92 |
| Age ≥ 35 years old | 113.62 | 50.40 | 189.00 | 87.06 | 104.45 | 114.10 | 124.95 | 143.86 |
| Mg (mmol/L) | 1.42 | 0.73 | 2.25 | 1.11 | 1.27 | 1.40 | 1.57 | 1.84 |
| Age < 30 years old | 1.41 | 0.73 | 2.25 | 1.11 | 1.26 | 1.39 | 1.56 | 1.84 |
| 30 ≤ Age < 35 years old | 1.42 | 0.89 | 2.00 | 1.12 | 1.27 | 1.41 | 1.59 | 1.86 |
| Age ≥ 35 years old | 1.41 | 0.84 | 1.98 | 1.09 | 1.26 | 1.39 | 1.56 | 1.84 |
